# A Case Report on a Syndesmotic Ankle Injury Treated With Arthrex TightRope

**DOI:** 10.7759/cureus.67185

**Published:** 2024-08-19

**Authors:** Amit Kale, Vishal S Patil, Abhishek Nair, Mohammed Talha Muneer

**Affiliations:** 1 Orthopaedics, Dr. D. Y. Patil Medical College, Hospital, and Research Centre, Dr. D. Y. Patil Vidyapeeth (Deemed to be University), Pune, IND

**Keywords:** road traffic accident (rta), trimalleolar fracture, pronation external rotation injury, syndesmotic ankle injury, arthrex tight rope

## Abstract

Syndesmotic ankle injuries, often referred to as "high ankle sprains," pose intricate challenges in orthopedic practice, particularly among athletes engaged in high-impact sports. Conventional treatments have encompassed conservative approaches and the use of syndesmotic screws, each beset by inherent limitations. The Arthrex TightRope system has emerged as a pioneering alternative, heralded for its capacity to facilitate physiologic micromotion, eliminate the necessity for hardware removal, and expedite early rehabilitation.

This case report delineates the management of a 29-year-old male professional soccer player who suffered a trimalleolar ankle fracture compounded by a severe syndesmotic injury subsequent to a road traffic accident. The patient underwent a comprehensive treatment involving open reduction and internal fixation (ORIF) of all three malleoli, complemented by syndesmotic stabilization employing the Arthrex TightRope system. Post-operative care encompassed a regimen of gradual weight-bearing and methodical rehabilitation.

At the one-year follow-up, the patient demonstrated excellent ankle joint function devoid of pain or complications related to hardware, underscoring the efficacy of managing syndesmotic and malleolar fractures successfully. This case underscores the potential advantages of integrating traditional ORIF techniques with contemporary syndesmotic fixation strategies like the TightRope system for complex ankle fractures, advocating for further research to refine their optimal utilization in clinical settings.

## Introduction

Syndesmotic ankle injuries, commonly known as "high ankle sprains," are complex injuries that can significantly impact an athlete's performance and career longevity. These injuries account for approximately 10% of all ankle injuries and are particularly prevalent in contact sports such as soccer, rugby, and American football [1]. The syndesmosis, a fibrous joint comprising four ligaments, plays a crucial role in maintaining ankle stability during weight-bearing activities. Disruption of this structure can lead to chronic instability, prolonged recovery times, and decreased athletic performance if not managed appropriately [2].

Traditional treatment methods for syndesmotic injuries have included conservative management for low-grade sprains and screw fixation for more severe cases requiring surgical intervention [3]. However, rigid screw fixation has several drawbacks, including the need for a second surgery to remove the hardware, potential for screw breakage, and limited physiological movement of the syndesmosis during the healing process [4].

In recent years, the Arthrex TightRope system has emerged as an innovative alternative for syndesmotic fixation. This device consists of a braided, non-absorbable suture loop tensioned between metallic buttons placed on the outer cortices of the tibia and fibula [5]. The TightRope system offers several potential advantages over traditional screw fixation:

(a) It allows for physiologic micromotion of the syndesmosis, which may promote more natural healing.

(b) It eliminates the need for routine hardware removal, reducing the risk associated with a second surgery [6].

(c) It enables earlier weight-bearing and rehabilitation, potentially leading to a faster return to sport [6].

Despite these advantages, the optimal management of syndesmotic injuries remains a topic of debate in the orthopedic community. While numerous studies have reported favorable outcomes with the TightRope system [7,8], long-term comparative studies with traditional techniques are still limited.

This case report presents a professional soccer player with a high-grade syndesmotic injury treated using the Arthrex TightRope system. By detailing the diagnosis, surgical technique, rehabilitation protocol, and outcome, we aim to contribute to the growing body of evidence supporting the use of this device in high-demand athletes. Furthermore, this case highlights the potential for an expedited return to sport following syndesmotic injury when managed with contemporary fixation techniques and an appropriate rehabilitation program.

## Case presentation

Patient information and presenting complaints

A 29-year-old male presented to the emergency department following a road traffic accident (RTA) with severe left ankle pain and deformity. The patient had no significant past medical history and was in excellent physical condition prior to the injury.

Physical examination

**Figure 1:**
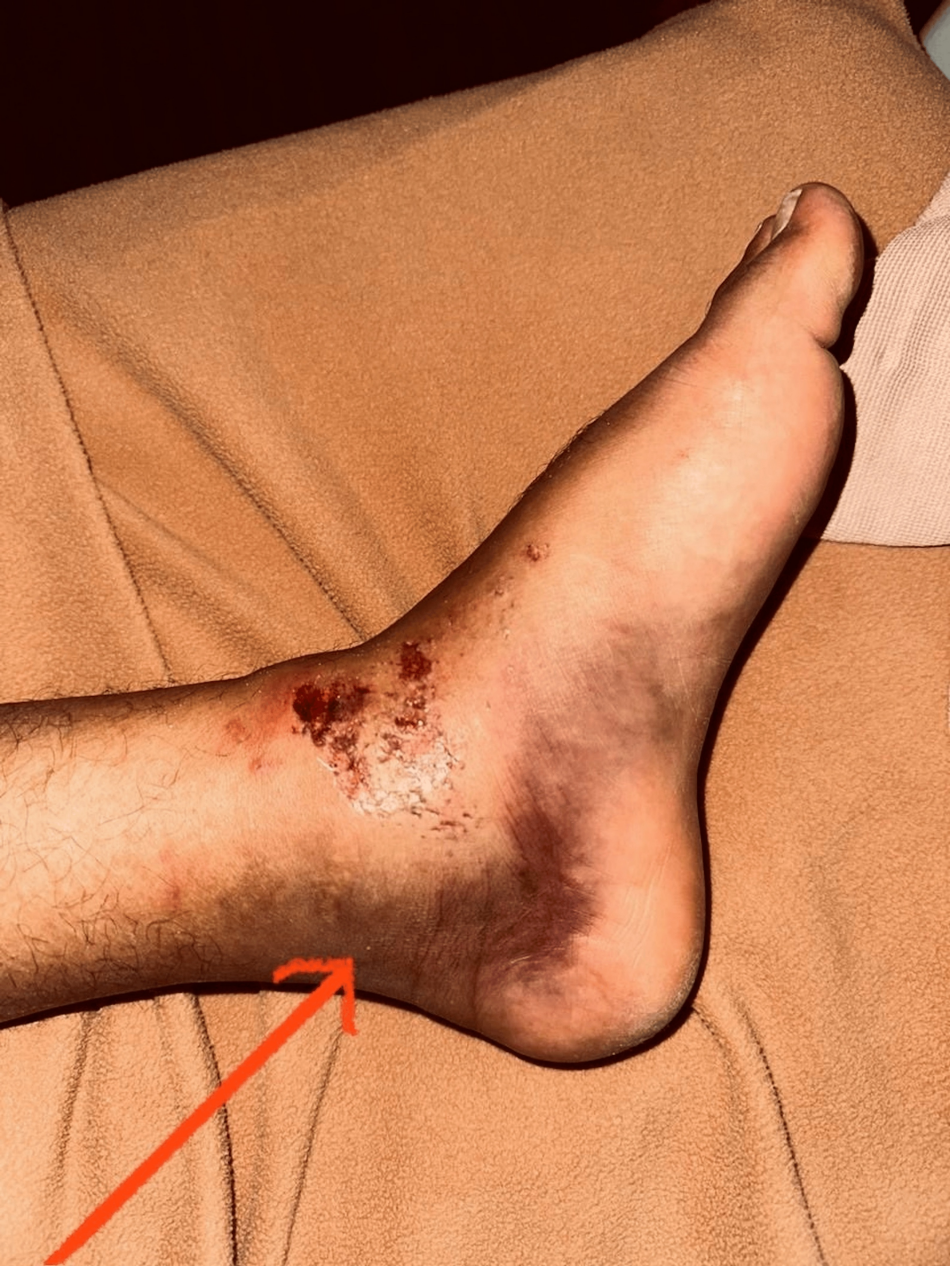
Clinical image of left ankle showing skin condition of the patient's ankle Red arrow shows the presence of swelling in the ankle.

Upon presentation, the patient was in visible distress due to pain. The left ankle showed significant swelling, particularly around the lateral malleolus and anterior aspect of the distal tibiofibular joint (Figure 1). There was marked tenderness on palpation over both the anterior and posterior aspects of the distal tibiofibular joint. The squeeze test, performed by compressing the tibia and fibula at mid-calf level, elicited pain in the syndesmotic region, indicating a positive result. The external rotation stress test, which involved stabilizing the tibia and applying an external rotation force to the foot, also produced pain in the syndesmotic region and was considered positive.

Despite the severity of the injury, the neurovascular status of the foot was intact. The patient had normal sensation in all dermatomes of the foot and ankle, and capillary refill in the toes was less than 2 seconds. Pedal pulses were palpable and symmetric compared to the uninjured side. Active range of motion was severely limited due to pain, but passive range of motion, although painful, did not reveal any gross instability of the talocrural joint.

Imaging studies

**Figure 2:**
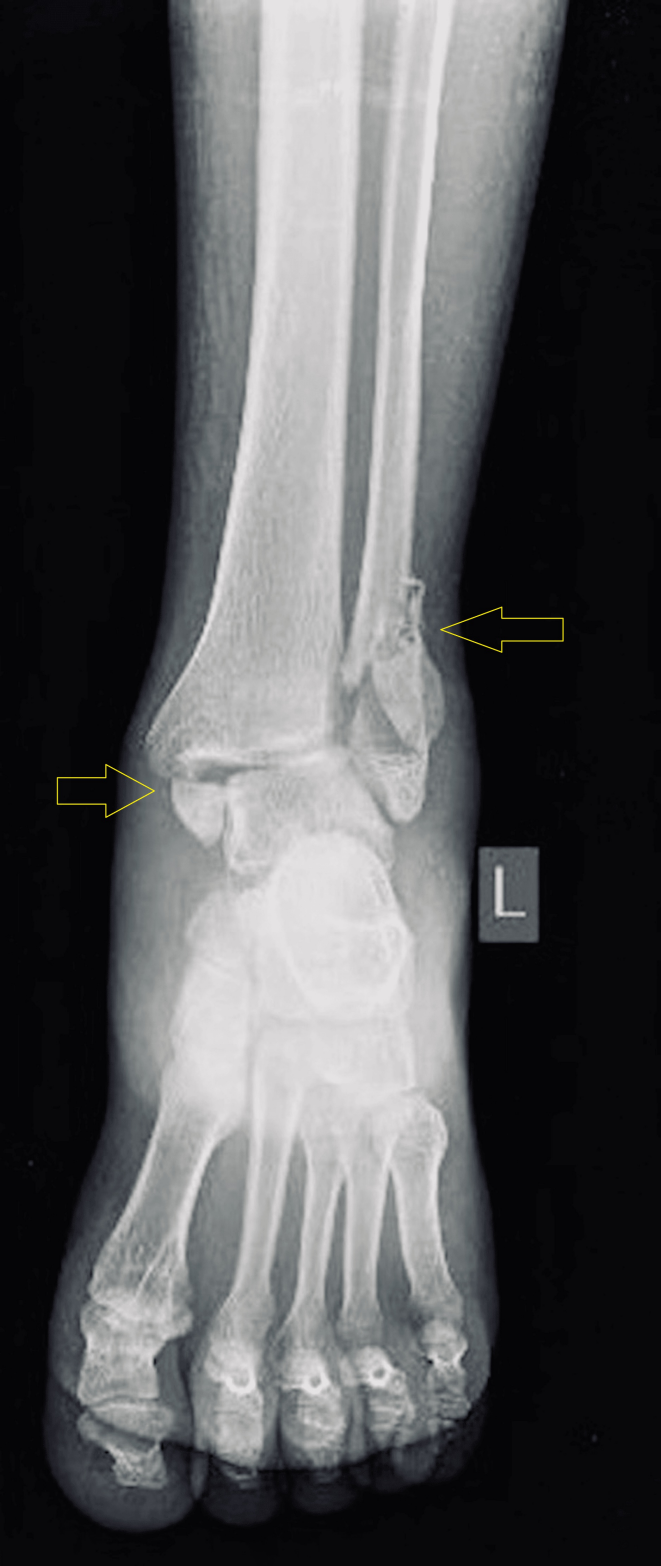
Pre-operative X-ray (anteroposterior (AP) view) of the left ankle.

**Figure 3:**
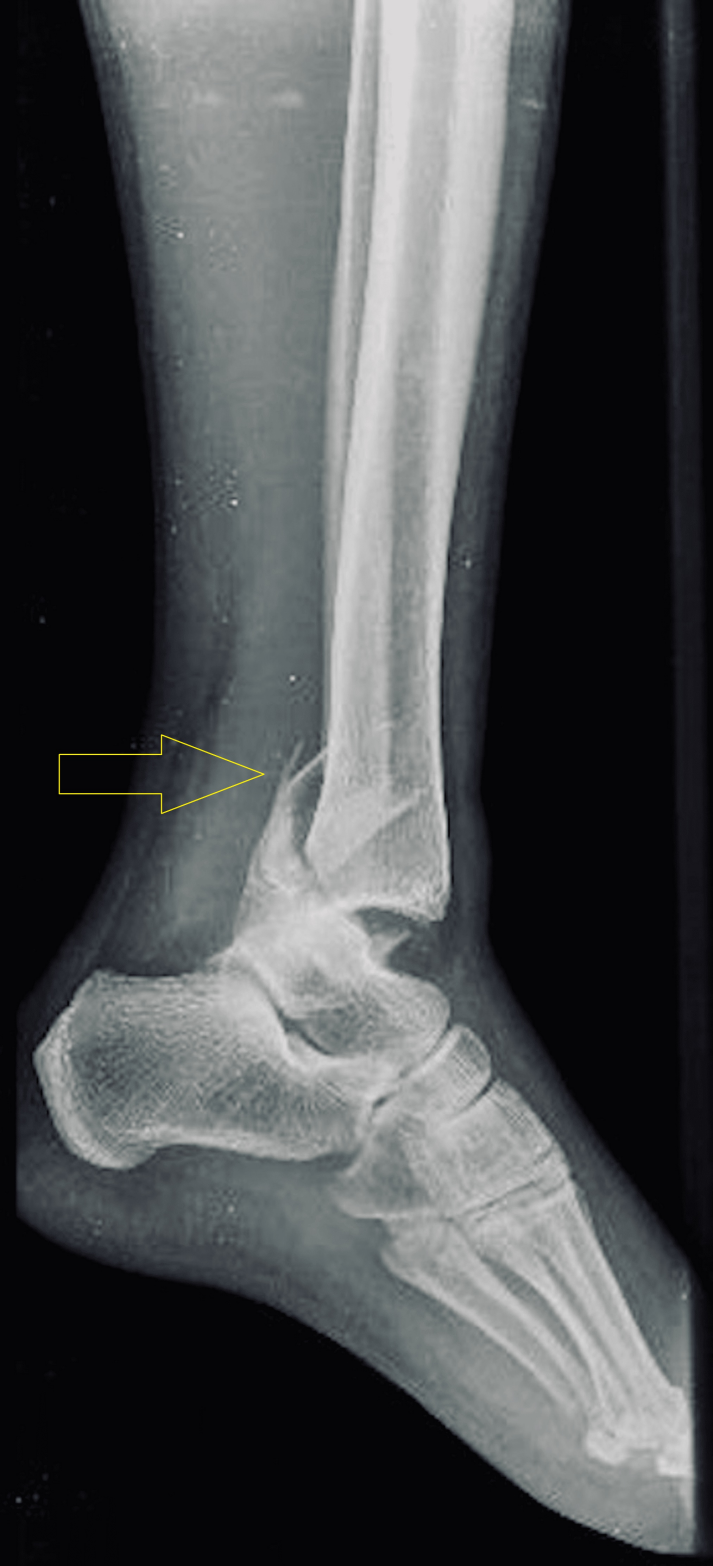
Pre-operative X-ray (lateral view) of the left ankle.

**Figure 4:**
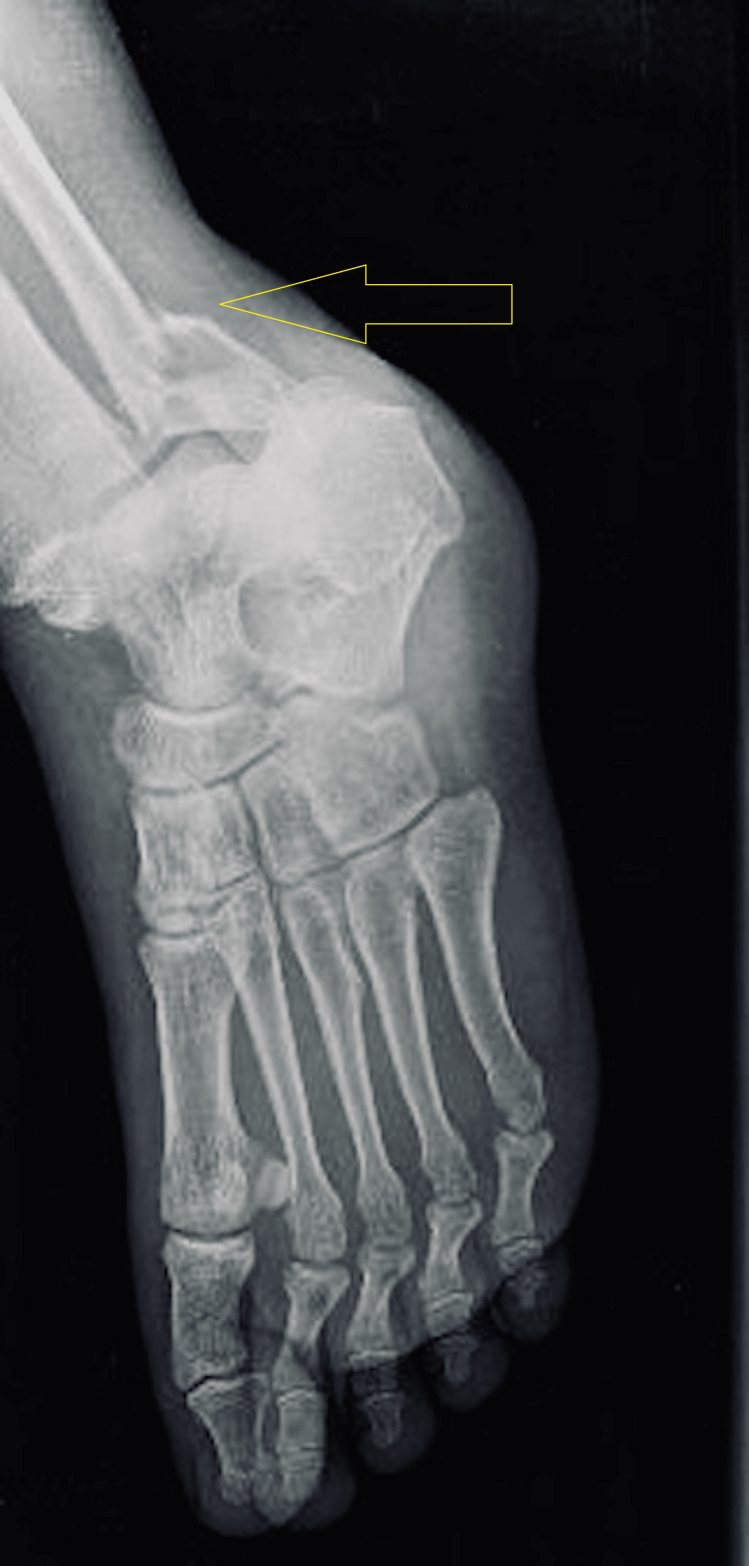
Pre-operative X-ray (mortise view) of the left ankle.

Initial radiographs included anteroposterior (AP), lateral, and mortise views of the left ankle (Figure 2, 3, 4). The AP and mortise views revealed the widening of the tibiofibular clear space, measuring greater than 6 mm, which is considered abnormal and suggestive of syndesmotic injury. Additionally, there was increased tibiofibular overlap on the anteroposterior view, further supporting the diagnosis of syndesmotic disruption. Radiographs revealed a trimalleolar fracture of the left ankle, involving the medial, lateral, and posterior malleoli. According to the Lauge-Hansen classification, the injury pattern was consistent with a pronation-external rotation type.

Given the high suspicion of a significant soft tissue injury, magnetic resonance imaging (MRI) was ordered. The MRI revealed a complete tear of the anterior inferior tibiofibular ligament (AITFL) and a partial tear of the posterior inferior tibiofibular ligament (PITFL). The deltoid ligament appeared intact, and there was no evidence of osteochondral lesions or other intra-articular pathology.

Diagnosis

Based on the mechanism of injury, physical examination findings, and imaging studies, the patient was diagnosed with a left trimalleolar ankle fracture with an associated high-grade syndesmotic ankle injury, specifically a complete tear of the AITFL and partial tear of the PITFL.

Treatment plan

After discussing the options with the patient, including traditional screw fixation and newer suture-button devices, the decision was made to proceed with fixation of all three malleoli with syndesmotic fixation using the Arthrex TightRope system. This choice was based on the potential benefits of allowing for physiologic micromotion of the syndesmosis during healing, avoiding the need for hardware removal, and the possibility of earlier rehabilitation.

Surgical procedure

The surgery was performed under appropriate anesthesia with the patient positioned supine on the operating table. After standard prepping and draping, the procedure began with the lateral malleolus. A direct lateral approach was utilized to expose the fibular fracture. After achieving anatomic reduction, the fracture was fixed using a bridge plating technique. A lag screw was also placed to provide interfragmentary compression and enhance stability. This combination of bridge plating and lag screw fixation ensured robust fixation of the lateral malleolus.

The medial malleolus fracture was addressed next, the fracture was anatomically reduced. Fixation was achieved using two cancellous screws, ensuring stable fixation of the medial malleolus.

The posterior malleolus was then addressed using a posterolateral approach. Great care was taken to identify and protect the sural nerve during this part of the procedure. The approach was made between the tendo-Achilles and the fibula, providing an excellent exposure of the posterior fragment (Volkmann's fragment). A cancellous screw with a washer was used to stabilize this fragment, effectively reducing and fixing the posterior malleolus.

After addressing all three malleoli, the final step was to ensure syndesmotic stability. Despite the rigid fixation of the malleoli, it was decided to provide additional syndesmotic stabilization to prevent potential complications such as screw loosening or breakage often associated with traditional syndesmotic screw fixation. For this purpose, the Arthrex TightRope system was employed. A transverse tunnel was created across the level of distal tibia and fibula, and the TightRope device was passed through this tunnel. The device was then tensioned appropriately with the help of metal buttons on the distal medial tibial cortex and distal lateral fibular cortex to achieve and maintain anatomic reduction of the syndesmosis.

Throughout the procedure, intraoperative fluoroscopy was utilized to confirm adequate reduction and proper positioning of all hardware. After copious irrigation, the wounds were closed in layers, and a sterile dressing was applied. The ankle was then immobilized in a well-padded splint in a neutral position.

Post-operative care and rehabilitation

**Figure 5:**
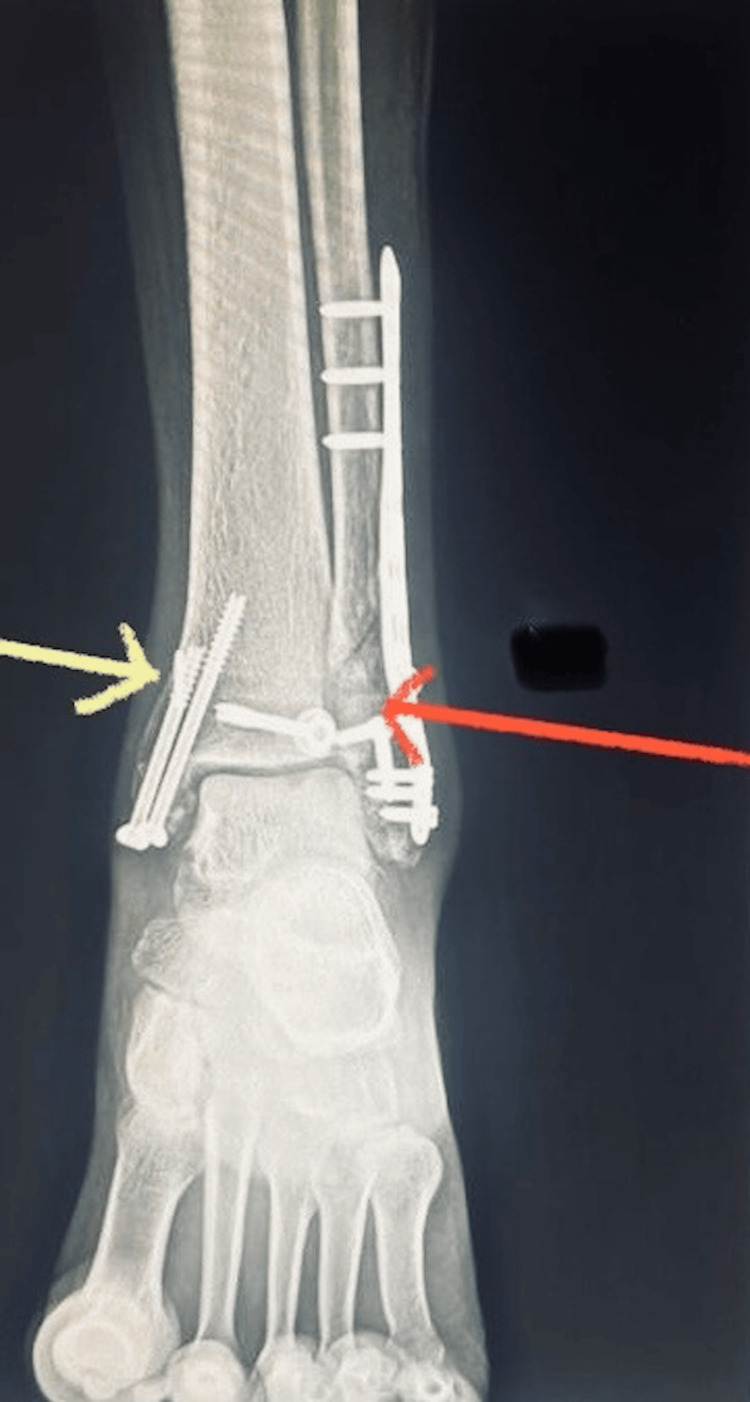
Post-operative X-ray (anteroposterior view) of the left ankle. Yellow arrow indicates the metal button medial distal tibial cortex and red arrow indicates the transverse lucent tunnel used for Arthrex TightRopes.

**Figure 6:**
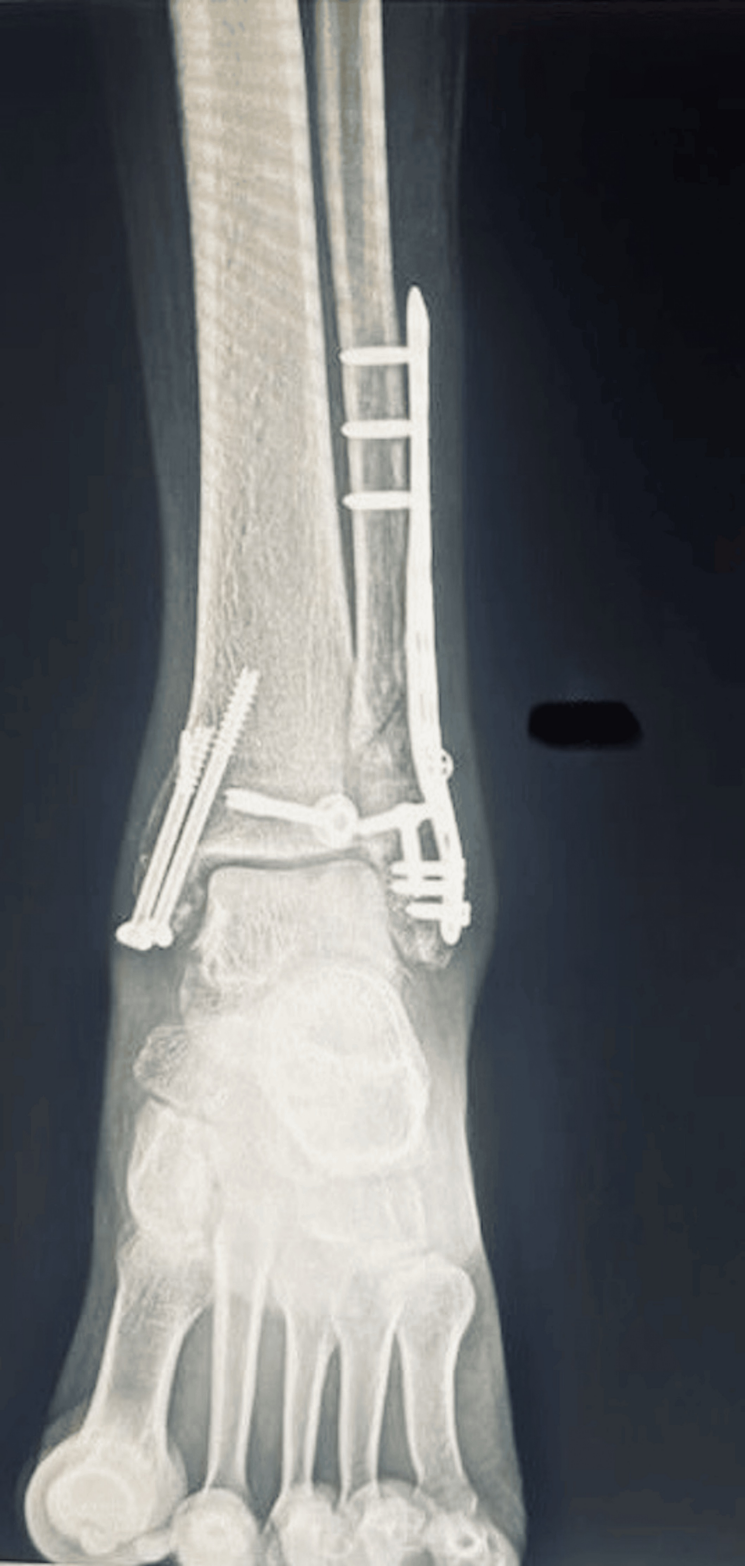
Post-operative X-ray (anteroposterior (AP) view) of the left ankle

**Figure 7:**
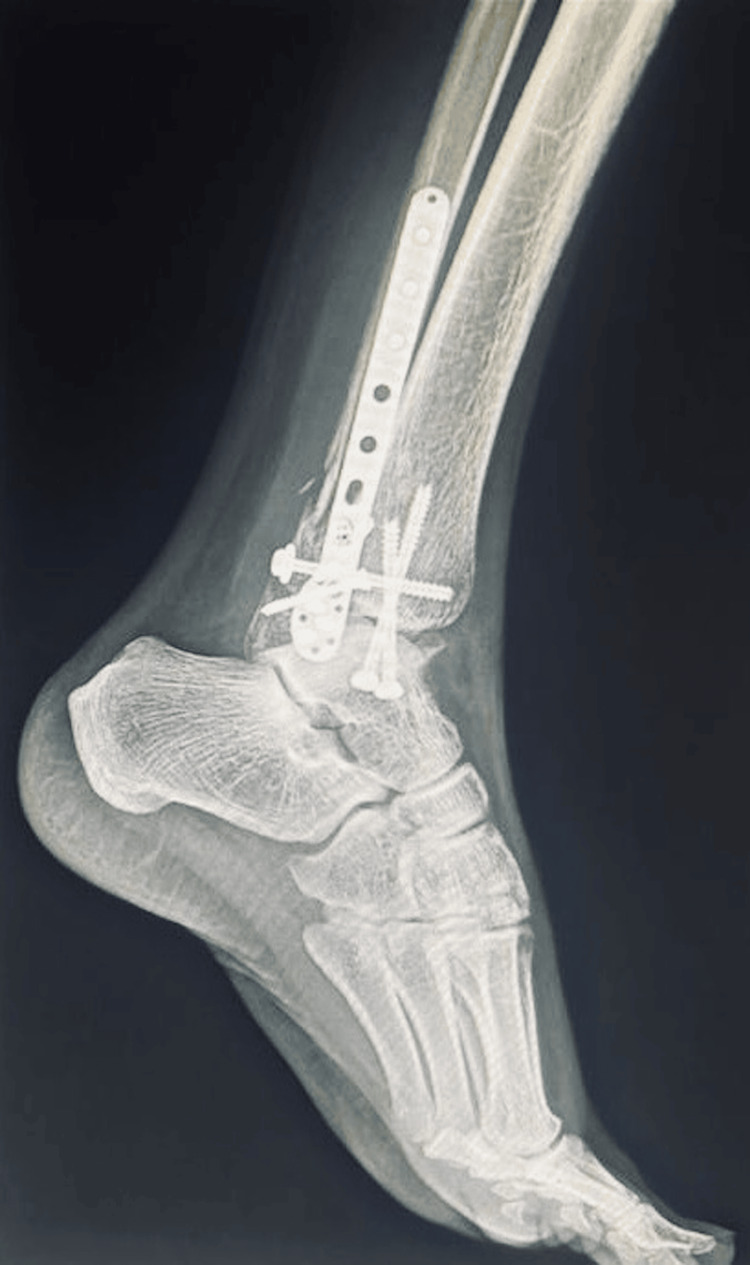
Post-operative X-ray (lateral view) of the left ankle.

**Figure 8:**
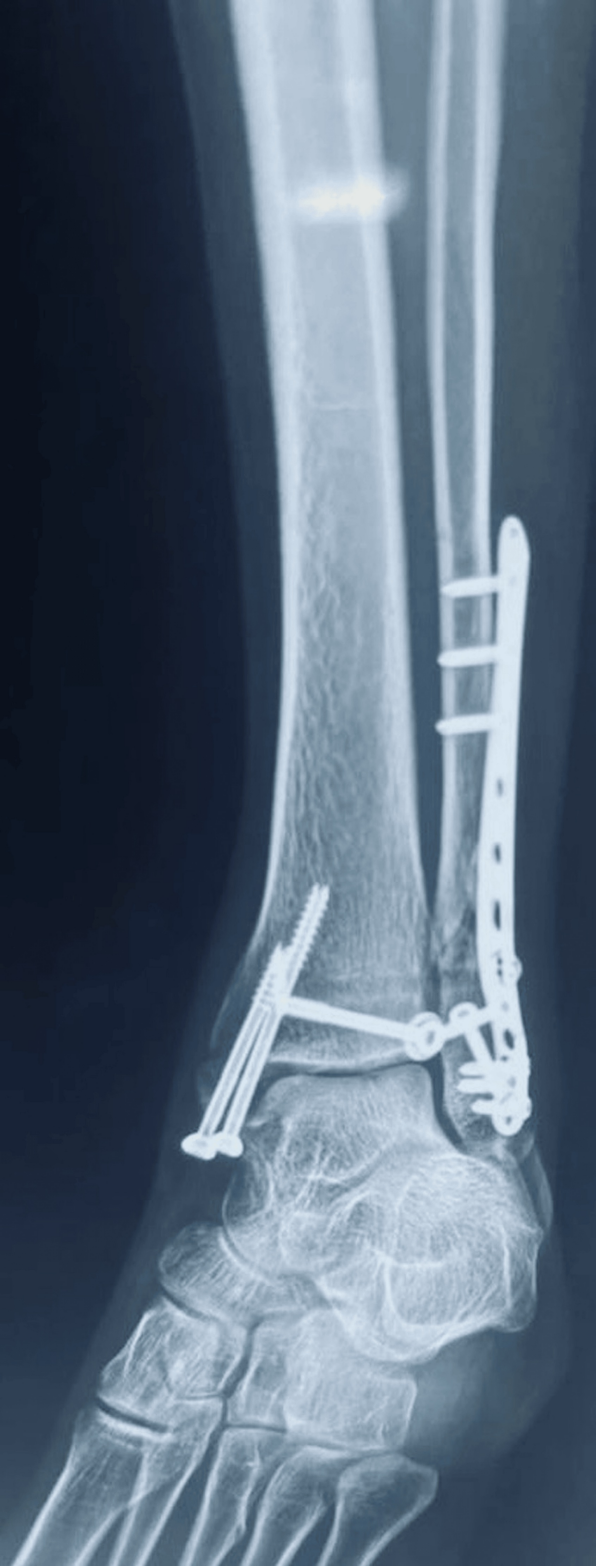
Post-operative X-ray (mortise view) of the left ankle.

Post-operative radiographs showed a transverse lucent tunnel across the syndesmosis with metal buttons along the distal tibial and fibular cortices (Figure 5), confirming the correct placement of the TightRope system (Figures 6, 7, 8). The patient was immobilized in a cast and instructed for non-weight-bearing ambulation for the initial post-operative period.

Progressive weight bearing was initiated at six weeks post-surgery. The patient was allowed to start full weight bearing at this time, which is earlier than typically allowed with traditional syndesmotic screw fixation. A structured rehabilitation program was implemented, focusing on regaining range of motion, strength, and proprioception.

Follow-up and outcome

At the one-year follow-up, the patient demonstrated a good range of motion in the ankle joint. He reported no pain or hardware discomfort during daily activities or light exercise. Radiographs showed a maintained reduction of the ankle mortise and healing of all malleolar fractures.

## Discussion

This case report demonstrates the successful management of a complex trimalleolar ankle fracture with syndesmotic injury using a combination of traditional open reduction and internal fixation (ORIF) techniques supplemented by the Arthrex TightRope system. The positive outcome, characterized by a good range of motion and absence of pain or hardware discomfort at one-year follow-up, aligns with growing evidence supporting the use of suture-button devices in syndesmotic injuries.

Our approach of using the TightRope system as an adjunct to traditional ORIF techniques is consistent with recent trends in the management of complex ankle fractures. Schepers et al. (2012) conducted a systematic review comparing suture-button devices with syndesmotic screw fixation, finding that suture-button fixation resulted in an earlier return to work and sports, as well as a lower rate of implant removal [4]. Our patient's ability to begin full weight-bearing at six weeks post-operation supports these findings, suggesting that suture-button devices may indeed allow for an earlier rehabilitation.

The decision to use the TightRope system in addition to comprehensive ORIF of all three malleoli was based on the desire to provide extra stability and mitigate the common complications associated with traditional syndesmotic screw fixation. Our patient's good functional outcome at one year, with no reported hardware issues, aligns with these biomechanical advantages.

Kortekangas et al. (2015) conducted a prospective randomized study comparing TightRope and syndesmotic screw fixation, finding that the TightRope system more accurately maintained syndesmotic reduction as assessed by bilateral computed tomography [6]. While our case report lacks long-term radiographic follow-up, the maintained reduction and good functional outcome at one year are promising and consistent with these findings.

The absence of hardware-related complications in our case is particularly noteworthy. Andersen et al. (2015) reported a high complication rate after syndesmotic screw removal, including infection and recurrent diastasis [7]. By using the TightRope system, we avoided the need for hardware removal, potentially reducing the risk of these complications. This advantage is especially relevant in high-demand patients or athletes where early return to activity is crucial.

However, it's important to note that our case report represents a single patient experience. While the outcome was positive, larger studies such as Inge et al. (2016) have shown mixed results when comparing dynamic (suture-button) versus static (screw) distal tibiofibular fixation [8]. They emphasized the need for high-quality randomized controlled trials to definitively establish the superiority of one method over the other.

The use of the TightRope system in conjunction with ORIF for all three malleoli in a trimalleolar fracture is less commonly reported in the literature. Most studies focus on isolated syndesmotic injuries or Weber C fibular fractures. Our case suggests that this combined approach may be beneficial in complex ankle fractures with syndesmotic involvement, but further research is needed to confirm this finding.

## Conclusions

In conclusion, while our case report adds to the growing body of evidence supporting the use of suture-button devices in syndesmotic injuries, it also highlights the need for larger, long-term studies specifically addressing complex ankle fractures with syndesmotic involvement. Future research should focus on comparing outcomes of traditional ORIF with and without suture-button augmentation in trimalleolar fractures to better define the role of these devices in complex ankle trauma.
